# Lessons learned: program messaging in gender-transformative work with men and boys in South Africa

**DOI:** 10.3402/gha.v8.27860

**Published:** 2015-09-07

**Authors:** Amanda P. Viitanen, Christopher J. Colvin

**Affiliations:** 1 Global Health Sciences, University of California–San Francisco, San Francisco, CA, USA; 2 School of Public Health and Family Medicine, University of Cape Town, Cape Town, South Africa

**Keywords:** health systems and gender, health determinants, Africa, health intervention

## Abstract

**Background:**

Adherence to traditional notions of masculinity has been identified as an important driver in the perpetuation of numerous health and social problems, including gender-based violence and HIV. With the largest generalized HIV epidemic in the world and high rates of violence against women, the need for gender-transformative work in South Africa is broadly accepted in activist circles and at the national and community level. Because of the integral role men play in both of these epidemics, initiatives and strategies that engage men in promoting gender equality have emerged over the last decade and the evidence base supporting the effectiveness of masculinities-based interventions is growing. However, little research exists on men's receptivity to the messages delivered in these programs.

**Objective:**

This article examines the current practices among a set of gender-transformation initiatives in South Africa to see what lessons can be derived from them. We look at how South African men participating in these programs responded to three thematic messages frequently found in gender-transformative work: 1) the ‘costs of masculinity’ men pay for adherence to harmful gender constructs; 2) multiple forms of masculinity; and 3) the human rights framework and contested rights.

**Design:**

This article synthesizes qualitative findings from in-depth interviews, focus group discussions, and ethnographic research with men participating in several gender- and health-intervention programs in South Africa. The data were collected between 2007 and 2011 and synthesized using some of the basic principles of meta-ethnography.

**Results and conclusions:**

Overall, men were receptive to the three thematic messages reviewed; they were able to see them in the context of their own lives and the messages facilitated rich dialog among participants. However, some men were more ambivalent toward shifting gender notions and some even adamantly resisted engaging in discussions over gender equality. More research is needed to gauge the long-term impact of participation in interventions that target gender and health.

Deep and persistent gender inequalities, patriarchal gender ideologies, and norms around masculinity, in particular, have been identified as critical factors in addressing a wide range of health and social problems in South Africa, including the closely entwined epidemics of HIV/AIDS and sexual violence (1–3). There have been a number of state, civil society, and community-driven efforts to transform gender practices and ideologies, and many of these initiatives are beginning to make conscious efforts to involve men and boys in their programing. Research around the contribution of masculinity to these health and social issues and the potential for changes in gender norms is also gaining momentum.

Interventions and research programs that focus on masculinities and health have taught us a great deal about the conventional ideas, and experiences of masculinity in South Africa – as well as the relational nature and multiplicity of masculinities found in South African society (4, 5). They have also highlighted many of the more destructive effects of adhering to rigid gender roles, and preliminary research suggests that once aware of these ‘costs’ and their impact, men are willing to change (6, 7).

Evidence supporting the effectiveness of masculinities-based programing in prompting behavior change is growing and a core set of programmatic messages (including those covered in this article) is becoming increasingly common in these programs. However, what is currently missing from the literature is closely observed research on the mechanisms of change that might connect the delivery of program messaging content to its reception by participants, and to intended behavior change (2, 8, 9). This article seeks to contribute to filling this gap by synthesizing findings from a variety of research projects conducted on masculinities-focused interventions in South Africa. We are particularly interested in this first step of the process of behavior change, from messaging content design and delivery to participant reception and interpretation. We review ways that some widely recognized key concepts and messages, used by community and civil society actors in their efforts to shift gender norms and practices, are received, interpreted, transformed, or resisted by men participating in these programs.

## Gender transformation in South Africa: contexts and interventions

The need for gender-transformative work in South Africa is broadly accepted in state, academic, community, and activist circles (10–12). Although gender norms do vary by age, ethnicity, class, and geography, the dominant masculine ideals of toughness, social domination, physical strength, and sexual prowess (often through unprotected sex) are widespread (11). Harmful beliefs such as male sexuality being an uncontrollable force capable of overwhelming any intentions of safe sexual practices are widely reinforced by both genders (11). Risky sexual behaviors, including multiple concurrent partnerships, unprotected sex, and sexual conquest, are often described not merely as effects of male sexuality but as defining features (1). This is especially problematic in an environment with a generalized hyperendemic HIV epidemic (13).

Another common facet of conventional masculinity – an entitlement to exert dominance over others, and specifically, over women – is a key driver in another nationwide epidemic plaguing South Africa: gender-based violence (GBV) (14). Although there are important debates over whether the legitimation of male physical dominance is indeed ‘traditional’ and pervasive in local communities (5, 15), there is strong evidence that connects support for these patriarchal prerogatives to the perpetration of violence (16). The growing evidence base, from both developing and developed countries, supporting the direct relationship between exposure and perpetuation of GBV and increased risk of HIV infection also confirms the destructively synergistic nature of these closely linked epidemics (2, 3, 17). Gender norms that perpetuate gender inequality and GBV toward women are pervasive in many communities as well as the public sphere (18–20). A series of GBV indicator studies carried out in four of South Africa's nine provinces gauged the prevalence of GBV and found that of those women interviewed, 77% in Limpopo (21), 51.3% in Gauteng (22), 39% in Western Cape (23), and 37% in KwaZulu-Natal (24) had experienced some form of GBV (either within or outside of an intimate relationship) in their lifetimes. Similarly, the incidence of rape in South Africa, though difficult to approximate because of under-reporting and cultural normalization of physical and sexual violence (25), is widely considered to be at epidemic levels. A cross-sectional study conducted in three districts in KwaZulu-Natal and the Eastern Cape by the South African Medical Research Council found that 27.6% (466/1,686) of men admitted to raping a woman; one-fifth of those that had committed rape had done so with someone other than an intimate partner (i.e. an acquaintance, stranger, family member); and more than half those that had previously committed rape (53.9%) had done so more than once (26). Furthermore, perpetrators of GBV are more likely to engage in any of the following risky sexual behaviors: substance use, transactional sex and prostitution, and less frequent condom use (27–31).

These gender norms, not surprisingly, have direct, health consequences on men as well as on women (32). Men conforming to traditional notions of masculinity are more likely to contract a sexually transmitted infection, view sexual partners as adversaries, have more negative attitudes toward condom use, and as a consequence, use condoms less frequently (33–35). Many South African men are also reluctant to make use of healthcare services, believing that seeking health care shows signs of personal weakness (36). They represent only one-fifth of those who get tested for HIV and only 30% of those accessing life-saving antiretroviral treatment (ART) (33, 37).

The majority of existing gender equality programing works with women to address gender disparities (38). Empowering women and protecting their rights are essential objectives to any efforts to eliminate gender inequality. However, those involved in gender-transformative work have become increasingly concerned that efforts to defend women's rights portray the beliefs and behaviors of men as the ‘problem’ in need of a solution (39). Although in some ways, this diagnosis seems intuitive, even obvious; interventions that pathologize and reify men and masculinity oversimplify the complex, diverse terrain of men's experiences and practices, and can significantly reduce men's desire to accept these messages and participate in gender equity and health initiatives. Furthermore, programs operating under the notion that all men are in positions of power, playing the role of oppressor, miss many men who do not identify with this masculinity and who may in fact be committed to changing rigid gender roles (36).

## Efforts to include men and boys in gender interventions

Initiatives and strategies that engage or encourage men to directly have an impact on violence against women have been recently summarized by Michael Flood into a six-level approach, which seeks first to educate, then to engage, and mobilize (39). The various levels of engagement in these initiatives are summarized in Table 1. The examples in the table are drawn from the case study evidence we synthesize in the Findings Section of this article. ‘Sonke’ is Sonke Gender Justice and ‘Khululeka’ is the Khululeka Men's HIV Support Group.

**Table 1:** Levels of engagement in work with men and boys

Level of engagement	Examples and goals
Individual education	Responsible fatherhood programs, youth mentoring. GOAL: individual recognition that violence can be prevented. EXAMPLE: ‘One Man Can’ workshops that educate participants about GBV and encourage critical reflection on each participant's own experiences and acts of GBV (Sonke)
Community education	Face-to face programs, social marketing, media strategies. GOAL: reach groups of people with information and resources. EXAMPLE: Door-to-door campaigns and ‘open-airs’ at shopping centers to raise awareness at HIV testing for men (Khululeka)
Providers/professionals education	Workplace-based prevention, resource provision, and technical assistance. GOAL: educate healthcare providers, teachers, police, and other professionals who can play an important role in promoting gender equality and advocating GBV prevention. EXAMPLE: Training with lay HIV counselors about the needs and perspectives of HIV-positive men accessing HIV care (Khululeka)
Engagement of communities	Community mobilization efforts, awards programs, linkage of violence to other community issues. GOAL: bring communities together. EXAMPLE: ‘Community Action Teams’ that coordinate community-led gender transformative programing (Sonke)
Changing practices	Change traditionally male-dominated groups (sports, social groups) perceptions as to acceptability of women-directed violence. GOAL: reshape societal norms. EXAMPLE: Coaching of local soccer team as a support group project aimed at shifting perceptions of masculinity among older boys (Khululeka)
Effecting policy and legislation	School anti-violence programs, country-specific laws protecting victims. GOAL: support of a healthy society that is violence-free. EXAMPLE: Country-level reviews across Southern Africa of health and GBV policies and the involvement of men and boys in policy and programing (Sonke)

Adapted from Flood (39).

Because of the relatively recent emergence of the masculinities field, literature documenting the impact of participation in gender programing is still limited, but the most effective types of gender programing are becoming more evident. A global evaluation of 58 gender interventions found that men's participation in gender-transformative programing (as opposed to gender-neutral or gender-sensitive programing) is more effective at prompting behavior change (6). Studies looking at short-term behavior change among men who participated in some well-known gender-transformative interventions (such as Institute Promundo's Project H) (48), Engender Health's Men as Partners Program (49), and Sonke's One Man Can (9) support the assertion that gender-transformative interventions can catalyze behavior change. In addition, consensus on which program messages are critical to include in effective masculinities-based interventions (including those reviewed in this article) are also becoming clearer (9). Much of the impact evidence is quantitative in nature (in the form of pre- and post-participation surveys) and focuses on outcomes rather than practice (50). What this article seeks to add to the field is to delve further into the mechanisms of gender-transformative programing and see how these messages are delivered and received from the perspective of those implementing and participating in the programs.

Given that gender constructs are socially reinforced and firmly entrenched in society, shifting gender norms and practices requires change in many different domains of social, economic, and political life (51). Although few organizations have the capacity and resources to intervene at all these levels, many of the more successful ones tackle at least two or more of these levels and try to understand and intervene on socially constructed gender norms, relations, and practices, using a multipronged, multileveled approach. Sonke's One Man Can (OMC) campaign is one such intervention.

OMC, Sonke's flagship program, is a gender equality and health intervention whose aim is to reduce the spread of HIV/AIDS and GBV. The campaign uses a human rights framework and masculinities-based approach to promote gender equality. The primary component of the campaign is participatory workshops, conducted with men and boys as well as in mixed groups with women and couples. These workshops provide ‘safe’ spaces for discussion and critical reflection on the topics of gender, human rights, women's rights, and masculinities. Typically, participation in short-term workshops results in short-term behavior change, but Sonke combines the workshops with longer term community engagement in the form of community action teams. In addition to these direct interventions, Sonke has an array of other complementary gender equality programing (public service announcements, media campaigns through radio and television, engaging community leaders) (52) and advocates for policy change at both the national level (pushing President Zuma, e.g., to develop a National Strategic Plan [NSP] for gender-based violence and incorporate more services that target men into the NSP for HIV/AIDS) and international level (as co-chair of the MenEngage Alliance) (53).

By contrast, Khululeka Men's Support Group is a community-based organization (CBO) in Cape Town that offers support for HIV-positive men. The members of Khululeka are Xhosa-speaking male residents of the Cape Town township of Gugulethu, most of whom are unemployed and only have a few years of formal education. They are all HIV-positive and most of them are on ART. Unlike Sonke, which is a relatively well-funded non-governmental organization (NGO) with a national profile and active set of programs, Khululeka is a small CBO with little steady funding and a chronic lack of resources and capacity in the group. They are, however, reflective of the kinds of CBOs that Sonke tends to partner with in communities for the OMC campaign and other initiatives.

Some of the work Khululeka engages in includes the kinds of community mobilizing and awareness raising described above as part of the OMC campaign. These include information tables at shopping malls, door-to-door campaigns, and slots on community radio stations. Khululeka also provides its members with a private, ‘safe space’ in the form of weekly support group meetings for its members, who are all men living with HIV/AIDS and dealing, in most cases, with the demands of ART and treatment adherence. This support group component attempts to support and engage with members in the long term, but Khululeka's work has been hampered by lack of professional facilitation skills and adequate funding. As a small CBO, it has been unsuccessful at establishing partnerships with other NGOs and CBOs and, in contrast to Sonke, has little interest in or capacity for political advocacy (41).

## Methodology

This article synthesizes findings from five research projects on men, masculinity, and gender transformation conducted in South Africa between 2007 and 2011. Findings from a number of these projects have already been published (2, 9, 18, 41–44). This study was designed to bring the findings of these individual studies together and determine broad patterns and common themes in the strategy and reception of gender interventions that work with men and boys. This synthesis is not intended to be representative of gender-transformation work in South Africa or of the body of research on this topic. Rather it aims to take advantage of our ability to revisit the original data from a range of different but related projects we have conducted over the last 8 years in order to reflect on and synthesize lessons emerging from this work.

The largest of these research projects was a process and impact evaluation of the OMC Campaign implemented by Sonke Gender Justice. The research was carried out by Christopher J. Colvin over 3 years (2008–2010) in six of South Africa's nine provinces and consisted of nine focus groups, 60 in-depth interviews, and 181 phone surveys. Participants in this evaluation are mostly black South Africans from low-income communities, OMC's target population. The campaign has targeted these communities (both rural and urban) because they are disproportionately affected by HIV (54).

The other major project was an ethnographic study of the Khululeka Men's Support Group. This study was conducted by Christopher J. Colvin and Steven Robins between 2007 and 2011. Research participants included members of the support group as well as community members, staff, and volunteers at other NGOs, and government officials who engaged with Khululeka.

We have also integrated data from smaller projects including an evaluation of the ‘Red Card Campaign’ against child sexual exploitation that Sonke undertook during the World Cup soccer tournament in 2010 as well as interviews conducted by Liese Pruitt and Benjamin Sieff with men and women in the community of Town Two, Khayelitsha, Cape Town on the topic of gender and health. Table 2 summarizes the various studies included in this synthesis.

**Table 2:** Summary of studies included in synthesis

Study	Summary of methodology and key sources
Evaluation of Sonke's ‘One Man Can’ campaign	Conducted between 2008 and 2010 in six of the nine South African provinces. Mixed methodology, including 9 focus groups, 60 in-depth interviews, and 181 phone interviews. Participants were primarily black South Africans in economically marginalized communities with high HIV prevalence (2, 9, 40)
Ethnography of Khululeka Men's Support Group	Conducted between 2007 and 2011. Ethnographic study with members of the Khululeka Support Group in Gugulethu, Cape Town, as well as with community members, NGO staff and volunteers, and government officials (18, 41–45)
Evaluation of Sonke's ‘Red Card’ campaign against child sexual exploitation	Conducted in 2010 and 2011. Process evaluation of Sonke and partner NGO's implementation of the Red Card campaign. In-depth interviews with 33 NGO staff and volunteers as well as community stakeholders in Cape Town and Johannesburg (46)
Short-term ethnographic studies of HIV, gender, rights, religion, and traditional healing in Town Two, Khayelitsha, Cape Town	Conducted in 2009 and 2010 with Benjamin Sieff and Liese Pruitt. Short-term ethnographic field research in Town Two, Khayelitsha, Cape Town with community members and NGO staff and volunteers on HIV, community mobilization, human rights, religion and traditional healing (47)

Ethical review for all projects was secured through the University of Cape Town's Faculty of Health Sciences Human Research Ethics Committee. Although each of these studies had their own set of specific research questions and objectives, they all used open-ended qualitative methods to collect data and were all concerned with the general problem of how to engage effectively with men in gender-transformation work. The similarities in focus across these studies, therefore, allowed for the extraction, comparison, and synthesis of findings.

To integrate findings from the studies, we used some of the basic principles of meta-ethnography (55). This is an approach to synthesizing qualitative data, usually in the context of systematic reviews. Because this study did not require identifying other research, we organized our analysis around the final four steps of this approach outlined by Noblit and Hare: 1) identifying relations between the studies, 2) translating them into one another, 3) synthesizing these translations into higher order interpretations, and 4) communicating the findings (56). In many ways, this approach reproduces the standard analytic techniques used in primary qualitative research, including constant comparison, checking for contrasting or contradictory cases, and developing higher order concepts and interpretations.

Unlike meta-ethnographies of published qualitative research, however, in this case, we had access to all of the original data for the original studies and the analysis process more closely approximated primary qualitative data analysis. The analysis process was also shaped by the fact that one of the authors (Colvin) had conducted or supervised all of the prior research and the other author (Viitanen) was new to the material. The first step of our joint analysis process was for both authors to review the published research (Table 2) and discuss emerging cross-cutting themes under the broad category of ‘lessons learned’ in gender-transformation work. This led to the identification of two broad areas of potential interest – program messaging and NGO strategies for engagement. We decided to focus on the question of messaging and identified several emergent subthemes under this theme. Viitanen then took these emergent subthemes and went back to the original interview and field note data and sought to clarify, refine, and extend these interpretations. Gaps and contradictory elements in the ongoing analysis process were discussed jointly and the original data were reviewed as necessary to provide further clarity. This combination of access to the original data and the involvement of a researcher who was new to the source material and able to test existing and offer alternative interpretations provided an important source of rigor to this synthesis.

## Findings

Our synthesis of the research findings reviews the content and reception of key programing messages used to engage men and boys in these interventions. The three thematic messages highlighted here are 1) the ‘costs of masculinity’ men pay for adhering to rigid and harmful gender constructs; 2) the existence of multiple forms of masculinity and differences and inequalities among men; and 3) applying a human rights framework when thinking about gender and contested rights. These messages are widely identified by gender activists and in the academic literature to be critical to effective gender-transformation programing. Our findings highlight the fact that although some aspects of these messages are embraced by the men (and women) involved in these programs; other aspects were elided, misunderstood, or even actively resisted by participants in ways that challenge the straightforward aims of gender-transformation initiatives. Our aim here is not to recommend alternative messages or to determine which messages are most effective or easily understood. Rather we aim to describe how these conventional messages are received, interpreted, and sometimes transformed or resisted by their intended recipients so that gender transformation programmers might have better insight into how and why the content of messages may (or may not) translate into changes in attitude and practice. For further background and analysis on many of the themes discussed below, please refer to the original sources cited in Table 2.

In the end, our findings suggest that messaging around the costs of masculinity was generally well-received and effective in reframing gendered perceptions, but notions of multiple masculinities and the link between human rights and gender rights were much more complicated in their reception. We also noted that program implementers and community participants sometimes shared the same reservations around messaging.

### The costs of masculinity

A core message of masculinities-focused programing is that men incur significant social and health costs (both directly and indirectly) as a consequence of their adherence to dominant forms of masculine identity and behavior (49, 57–59). One of the primary objectives of the OMC Campaign workshops is to familiarize men with these costs and help them realize how conforming to narrow and rigid gender constructs has negatively affected not only women around them but also their own lives and the lives of other men they know. Risky sexual behavior, physical conflict with other men, sexual and physical violence against women, drug and alcohol abuse, depression and other mental health issues, unhealthy interpersonal relationships, and poor health-seeking behavior are all addressed in the programing materials. The workshop modules are designed to help concretize these costs through role-playing activities, and honest group discussions, driven by questions aimed at self-reflection.

I attended a workshop about gender; it played an important role in my life understanding of gender roles and balance. The workshop focused on roles by men and women, the facilitator started with our family backgrounds. From our family backgrounds we found that there are so many men who grew up (as first-born) in their families. As a result, they had to do family chores like washing dishes, cleaning and cooking for their younger brothers and sisters. It was realized that most of we tend to stop doing those important chores immediately when we grow up or get married. We became shy from doing them because we thought they were supposed to be done by girls or women. Hence, when we get married, we expect our wives to do the work for us instead, whether the wife works or not. It just becomes their burden to cook, clean and wash dishes and laundry. The discussion at that workshop really made me to open my forever-closed eyes considering that I used to cook for my younger brothers as we were growing but now that I am married, I do not do any of those chores. That was one of my best sessions in the program. [Male OMC Workshop participant]

A number of Sonke staff and all workshop facilitators have participated in the OMC workshop trainings themselves and have spoken about the impact of this process of self-reflection and the revelations it can elicit. Some participants are former perpetrators of violence against women and have credited participation in this workshop (or similar gender-transformative programing) as the catalyst for their transformation. Sonke staff have posited that recognizing how conforming to hegemonic masculinity has adversely affected their own lives serves as a catalyst for desire to change.

Many of the participants of OMC workshops do indeed report identifying and responding strongly to these messages about the costs of masculinity. In particular, reluctance to seek medical care, social pressure to demonstrate sexual prowess, and the inability of many un- and underemployed men to fulfill social expectations to financially support their families generated rich discussion and debate across our studies. To help participants recognize examples from their own lives, facilitators share their personal experiences and past transgressions. This also fosters an environment in which it feels safe to share.

Although the members of the Khululeka Men's Support Group understand the costs of masculinity in broadly similar terms, they frame these costs and their responses to them in a slightly different way. Rather than tackling prevailing masculine norms and their effects directly in frank and challenging discussions, the way Sonke workshops operate, Khululeka has focused on creating safe spaces for HIV-positive men to disclose their injuries and vulnerabilities to each other. Many Khululeka members have come to believe that it was adherence to hegemonic norms of masculinity that resulted in their HIV-positive status. These men-only spaces (as opposed to OMC's often mixed workshop audience) create a forum for the men in Khululeka to reveal and discuss the challenges they face with respect to their illness, their relationships, their substance abuse, and their lack of employment (45). The conversations in these support group spaces are more diffuse, intimate, and open-ended, less directed by an outside facilitator or program, and less directly framed in terms of psychotherapeutic or human rights vocabularies. They talk freely and at length about the many ways in which the social norms of masculinity have cost them physically, psychologically, socially, and economically.

### Multiple masculinities

Multiple masculinity theory is a cornerstone of masculinity studies, and a component of the conceptual framework of many gender equality and health interventions that work with men and boys (60). The range of possible masculinities, both between and within communities in South Africa, is thus another key programing message. Across these various intervention programs, however, it appears difficult for differences between men to be recognized and accommodated within programing by both staff and participants. Indeed, this struggle to both recognize the diversity of men in South Africa and to translate it into effective programing has been a source of robust debate among Sonke staff – and among Sonke's other stakeholders such as other local NGOs and academic partners – and it can be seen on a number of levels in OMC workshop programing. At the organizational level, Sonke works as a gender justice network, with the OMC program being their flagship project for engaging men, and mobilizing change at the individual and community levels. In their mission statement and workshop programing, the target audience is defined as men and boys [implicitly all men and boys]. However, in practice, the communities in which workshops are conducted determine the target audience being reached. To date, the majority of workshop trainings have been carried out in predominately black South African communities (2, 61).

On a conceptual level, OMC programing is inclusive of the multiple masculinity theory and those who facilitate the workshops are well versed in this notion. Still, outside the workshops, discourse among participants often lapses into generalized descriptions of ‘men’ as being uniform in cultural upbringing, character, and by consequence, actions. This contradiction, of recognizing heterogeneity among men while simultaneously referencing to a singular masculine identity, was a recurring theme in interviews with OMC participants as well. In one focus group, for example, a number of HIV-positive young men, who provided home-based care to other sick men in their community, repeatedly asserted that ‘all men in their community’ (including themselves) behaved in a certain way and held certain ‘traditional’ beliefs. They spoke in great detail about how ‘men’ behave in sexual relationships, what they value about conventional gender roles, and how these gendered characteristics are not malleable but are rather the unchanging product of both culture and nature.

However, they did not seem to recognize that being sick and vulnerable themselves, seeking medical help through ART and HIV support groups, and providing intimate caring for others, were all conditions and behaviors that do not conform to the model of hegemonic masculinity they were busy describing and legitimizing. Instead, they seemed unable (or unwilling) to recognize the fact that they represented a significant shift away from these dominant norms and practices. The disconnect between discourse and reality in this example demonstrates how gender ideals and norms influence and organize thought and frame experience. It also illustrates that a great deal of creativity and flexibility with respect to gender norms may simply remain unrecognized.

Work with Khululeka members offered similar examples. Group members felt marginalized by their HIV status and most cited their divergence from ideals of hegemonic masculinity as the primary reason for joining Khululeka. Participants often compared being sick to being ‘infantilized’.

When you are HIV-positive, and on top of that unemployed you lose everything. Your wife and children don't respect you because you are sick, without a job and now you cannot provide for them. You are nobody, you are useless. This is why we have created Khululeka, to help men discover their manhood and dignity again. [Phumzile, founder of Khululeka]

The manhood Khululeka seeks to restore, however, is in some important ways a modified masculinity, one that incorporates many of the hegemonic ideals of male strength and dominance while avoiding those aspects of male identity that most compromise the health of men and their partners. Although there is more than occasional reference to women's rights and equality, as well as recognition of the place of non-heteronormative sexualities, in their discourse, the model of masculinity Khululeka promotes is left intact enough to remain recognizable and appealing to members. By remodeling hegemonic masculinity into a more positive and healthier version of its former self, men come to experience the support group space of Khululeka as one that does not explicitly challenge the broad outlines of a masculinity many still embrace even as they try and struggle to live up to its ideals.

Both Sonke and Khululeka have found it difficult to realize a fully fleshed-out model of multiple masculinities in their approaches, despite widespread recognition of this diversity in principle. Reference to non-dominant forms of masculinity, and in particular non-heteronormative sexualities, does often get discussed when Sonke and Khululeka members are addressing gender and sexual rights under the new constitution (see next section). However, for the most part, these alternative forms of masculinity are cast as marginalized. The idea of a ‘hegemonic masculinity’ – and, in particular, a hegemonic masculinity raced as ‘black’ – continues to implicitly structure much of the conversation and practice of these programs.

### The human rights framework and contested rights: children, women, and LGBT

One area of both Sonke's and Khululeka's work that consistently produced heated debate and discussion was that of human rights, and in particular, the ways human rights were interpreted and applied in South Africa with respect to children; women; and members of the lesbian, gay bisexual, and transgender (LGBT) community. Although both Sonke and Khululeka (albeit in different ways) utilize a human rights framework, interviews with participants and facilitators in both programs revealed an ongoing tension between the right-based discourse of gender equality and local cultural discourses of masculinity and social power.

In general, male participants in both interventions expressed frustration with gendered power shifts in both public and private spheres, citing the impact of these changes at the household (e.g. women's sexual and reproductive health rights; division of labor), community (e.g. women in the workplace), and societal level (e.g. the law) (2).When asked about the function and significance of human rights in relation to gender, a majority of the men interviewed who had not participated in an OMC workshop and were not involved with an HIV or gender-related NGO echoed the sentiment, ‘Now, women have all the rights’ (2). We also heard this sentiment, however, from OMC participants, Khululeka members, and other members of rights-oriented CBOs. Although many recognized that the South African Constitution is one of the most progressive and inclusive affirmations of universal human rights in the world, they also felt that its application translated into greater protection of what was framed as ‘women's rights’.

Across focus groups, men cited generic anecdotes (usually secondhand, with no names attached) to demonstrate this over-emphasis on ‘women's rights’. One recurring tale was that of a woman complaining to the police that her rights were being violated because of domestic violence and the police taking action and arresting the accused without due process. The story was often followed by echoes from other men that if the scenario were reversed (and it was a man who reported their partner to the police) they would not receive the same protection under the law, and in addition, would be mocked for even bringing the matter to the police (41).

Like I said before if an argument emerges between me and my wife the law will always protect the women than me. If it happens that in the middle of the argument she throws herself in a dangerous place, injure[s] herself and rush[es] out to the police station. I will not win that battle. She can always say that I have been abused by my man. Before human rights apply, already women rights take charge. I won't even have chance to explain, because I will be seen as someone who has not only violated human rights but human and women rights. [Male OMC Workshop participant]

In addition to legal implications, men expressed frustration at the household and occupational level and the disruptive impact they perceived that this shift is causing within the family unit. Historically, the gendered division of labor in South Africa has been fairly concrete. The increasing presence of women in the workforce (coupled with the existing high rate of unemployment) has disrupted traditionally defined gender roles and shifted dynamics within the household. The men in Khululeka, in particular, expressed feelings of disempowerment that they attributed to recent efforts to empower women and promote women's rights. As group founder, Phumzile Nywagi puts it, even in the realm of HIV support groups and volunteer opportunities to work as lay counselors, men felt that women in their community had ‘more support groups, more power and more jobs’ in the health sector than men (43).

Of course, not all of the men in our research opposed gender equality and women's empowerment so explicitly, and many reported embracing these ideals and changing their behavior as a result of their participation in masculinities-focused interventions. For these men, however, these changes were always set against the backdrop of broad and significant tensions between a post-apartheid political discourse that emphasized human rights and a traditionalist discourse of patriarchy and culture that emphasized male control over domestic and social life. Many of those participating in both OMC workshops and Khululeka meetings identified the government as the agent that had usurped their family autonomy by setting laws that protect women and children and undermine their prior authority.

Although women typically disagreed that their rights were too powerfully protected, the topic of children's rights is one area that men and women connected to both organizations seemed to agree on. These rights were often described as being inappropriately asserted within the household by an external and intruding state. They spoke of the negative consequences that a rights-based approach to child rearing was having for both individual discipline within families as well as social discipline and morality at the community level. As with the archetypal story of the woman who gets the police to lock up her partner over the weekend, men and women alike, across the country and across interventions, told the same story about children who had, for example, memorized the child abuse hotline number and threatened to call it every time a parent attempted to discipline them. They further complained that children have extended the notion of children's rights from freedom from physical discipline to also include freedom from any kind of discipline, chastisement, or control over their behavior.

A third contested rights domain within human rights in these interventions was the area of sexual rights. Sonke staff describe a situation of pervasive homophobia in South Africa and throughout sub-Saharan Africa as a whole. South Africa's constitution explicitly addresses sexual rights and prohibits discrimination on the grounds of sexual orientation or gender identity (62, 63). Despite legal recognition, however, LGBT rights are still constrained by stigma, discrimination, and widespread homophobia (64).

And yet, homophobia is visibly absent from much of the programing of both OMC and Khululeka. At the time, no OMC workshop activity directly addressed LGBT rights, stigma reduction, or homophobia. Many staff reported covering LGBT issues in workshops but expressed difficulty addressing these issues comprehensively. The same was true in Khululeka's support group meetings and other events. Neither intervention engaged in detail with LGBT rights in their discussions and activities.

When the topic did come up, staff and support group leaders often reported being able to do little in terms of challenging these norms. Participants in both OMC and Khululeka generally accepted and reinforced the broader discourse of homophobia. There was widespread agreement that heterodox sexualities were unnatural and immoral. Many participants did agree with the idea that this difference did not necessarily excuse physical violence or verbal abuse directed at members of this community, but there was little room for discussion of these sexualities as anything other than abnormal and threatening.

## Discussion

Previous studies have looked at the efficacy of masculinities-based programing in prompting behavior change in South Africa and consensus on critical messages to include in programing (including those covered in this article) is growing. Our findings broaden this evidence base by illuminating some of the details of the process of message delivery and highlighting variations in message reception and interpretation. A better understanding of these mechanics will help those working in gender-transformative programing to continue to refine their curricula.

Of the three key program messages highlighted in this article consensus was greatest with respect to the costs of masculinities argument. Members of Khululeka, Sonke staff, and OMC workshop participants seemed to struggle least with recognizing and translating this message in their own lives. In part, this may be because it names something about the crises of masculinity that many men are experiencing but do not have a frame for. Discussing gender identity in consequentialist (as opposed to normative) terms offers a way forward from rigid gender norms that are incompatible with their circumstances by validating a shift away from the *status quo*.

The multiple masculinities argument was recognizable when discussed theoretically but the ability to recognize the constructedness of gender norms had less traction. The influence of the hegemonic norm made it difficult to talk about anything except positive but relatively small modifications away from this ideal. It also made it difficult for people to recognize the diversity of masculinities in their own lives and communities or to accept that they fit anywhere outside of the hegemonic box.

It is well documented in the literature that there is often resistance to rights-based discourse, especially when it is perceived as being imposed by external political actors. The human rights discourse had traction in both groups when discussed at the societal level as far as issues of general fairness, the importance of tolerance, and the prohibition against physical violence and abuse. This was especially true when linked to political arguments about rights in the context of the struggle against apartheid. However, acceptance of these rights was more fragmented when individuals applied them at the household level and with respect to children's and sexual rights. In these contexts, rights discourses were viewed as externally imposed (largely by the state and western culture), and the appropriateness of enforcing them in the private sphere was widely questioned.

The lack of formal or confident inclusion of advocacy for the LGBT community in OMC programing was notable. One possible explanation for why this content area did not manifest as strongly is that within the public's perception of the larger human rights framework in South Africa, the inclusion of LGBT rights is relatively recent. Even some who identify as firm champions of human rights in political circles have yet to recognize and accept the rights of members of this community. The ongoing and pervasive homophobia in the country requires LGBT issues to be addressed as an explicit and central component of one's activism.

Some of the differences in perspective between Sonke staff and participants and Khululeka members are to an extent attributable to the different social, educational, and economic strata they represent. Although the men in both groups share a racial and linguistic history, and may even live in the same communities, many of Sonke's field staff have secondary and tertiary qualifications, speak English along with several other languages well, and are increasingly part of a class and cultural context rich in modernist discourses of human rights and gender equality (65). Khululeka members, by contrast, are for the most part unemployed, have little educational background, and live precarious economic lives.

Another important factor that accounts for some of the differences between these two groups is their difference in purpose. Sonke is an organization that focuses on a wide range of gender-related issues, whereas Khululeka is a men's HIV support group (18). While Sonke includes HIV as one of its core thematic foci and political concerns, its programing is designed and delivered as a broad gender transformation intervention rather than a health intervention. Khululeka, on the other hand, follows a more therapeutic logic, organizing its programing around ways to prevent and treat the disease, recognizing gender as an important cofactor in this health condition. Although its founder originally intended Khululeka to work within a gender transformation framework, and frequently used the language of gender transformation, Khululeka members often resisted this framing and pushed for gender work that might be more accurately considered gender sensitive. This is most evident in the group's adaptation of masculine ideals to be compatible with their HIV-positive status. Reframing health-seeking behavior as a means to reclaiming independence and self-reliance allows the men to adhere to dominant notions of masculinity by redefining how this can be achieved.

## Limitations

The synthesis developed in this article benefited from access to the original research data as well as the inclusion of a researcher new to the source data and thus able to offer fresh insights and test existing interpretations. As with any synthesis of existing data, however, we were limited in the extent to which we could probe and refine emerging cross-case analyses. In primary qualitative research, one refines the methods and data collection tools as one progresses and in this case, we did not have that opportunity. It should also be noted that this synthesis does not aim to capture the breadth of gender transformation work in South Africa. Rather, it aims to look across a set of closely observed and geographically proximate case studies to identify potential lessons learned and avenues for further research.

## Conclusions

This article adds to the growing evidence base supporting the utility of men's inclusion in efforts to transform gender constructs and men's receptivity to participating in such interventions. Our findings complement the existing literature on the impact and efficacy of gender-transformative programing (‘what works’ in gender transformation) by focusing on the process of program delivery (i.e. ‘how gender transformation works’). The objective was to identify those messages participants consistently identified with, either because they provide a framework through which they could recognize their lives and commitments, and/or because they generated complex debate, and to see how they were delivered in practice and how men responded. We are not suggesting that masculinities-based interventions alone can effectively achieve gender equality. In order for gender norms to change at a community or society level, a partnership of men and women focusing on the common goal of gender equality is required (66). Strategies regarding whether to do this separately and/or together is a topic that needs additional attention. Programs that capitalize on the synergies of men's and women's health gains facilitated by gender equality stand a better chance of successfully achieving sustainable and widespread gender transformation. Finally, further research needs to be done on long-term messaging retention and behavior change.
